# Replication of the association of chromosomal region *9p21.3 *with generalized aggressive periodontitis (gAgP) using an independent case-control cohort

**DOI:** 10.1186/1471-2350-11-119

**Published:** 2010-08-09

**Authors:** Florian D Ernst, Katharina Uhr, Alexander Teumer, Jutta Fanghänel, Susanne Schulz, Barbara Noack, Jose Gonzales, Stefan Reichert, Peter Eickholz, Birte Holtfreter, Peter Meisel, Gerard J Linden, Georg Homuth, Thomas Kocher

**Affiliations:** 1 Interfaculty Institute for Genetics and Functional Genomics, Department of Functional Genomics, Ernst-Moritz-Arndt-University Greifswald, Greifswald, Germany; 2 Department of Restorative Dentistry, Periodontology, and Endodontology, Unit of Periodontology, Ernst-Moritz-Arndt-University Greifswald, Greifswald, Germany; 3 Department of Operative Dentistry and Periodontology Martin-Luther-Universität Halle-Wittenberg, Halle-Wittenberg, Germany; 4 Department of Periodontology, Universitätsklinikum Carl Gustav Carus, Dresden, Germany; 5 Department of Periodontology, Justus-Liebig-University Giessen, Giessen, Germany; 6 Department of Periodontology, ZZMK (Carolinum), Johann Wolfgang Goethe-University Frankfurt, Frankfurt, Germany; 7 Centre for Public Health, School of Medicine, Dentistry and Biomedical Sciences, Queen's University, Belfast, Northern Ireland, UK; 8 Department of Medical Oncology, Erasmus MC, Rotterdam, The Netherlands

## Abstract

**Background:**

The human chromosomal region *9p21.3 *has been shown to be strongly associated with Coronary Heart Disease (CHD) in several Genome-wide Association Studies (GWAS). Recently, this region has also been shown to be associated with Aggressive Periodontitis (AgP), strengthening the hypothesis that the established epidemiological association between periodontitis and CHD is caused by a shared genetic background, in addition to common environmental and behavioural risk factors. However, the size of the analyzed cohorts in this primary analysis was small compared to other association studies on complex diseases. Using our own AgP cohort, we attempted to confirm the described associations for the chromosomal region *9p21.3*.

**Methods:**

We analyzed our cohort consisting of patients suffering from the most severe form of AgP, generalized AgP (gAgP) (n = 130) and appropriate periodontally healthy control individuals (n = 339) by genotyping four tagging SNPs (rs2891168, rs1333042, rs1333048 and rs496892), located in the chromosomal region *9p21.3*, that have been associated with AgP.

**Results:**

The results confirmed significant associations between three of the four SNPs and gAgP. The combination of our results with those from the study which described this association for the first time in a meta-analysis of the four tagging SNPs produced clearly lower p-values compared with the results of each individual study. According to these results, the most plausible genetic model for the association of all four tested SNPs with gAgP seems to be the multiplicative one.

**Conclusion:**

We positively replicated the finding of an association between the chromosomal region *9p21.3 *and gAgP. This result strengthens support for the hypothesis that shared susceptibility genes within this chromosomal locus might be involved in the pathogenesis of both CHD and gAgP.

## Background

Periodontitis is a complex inflammatory disease characterized by progressive destruction of the surrounding connective tissue and supporting alveolar bone of the teeth [1]. In adults older than 40 years, it represents the major cause of tooth loss [2]. For the most severe form of periodontitis, aggressive periodontitis (AgP), the prevalence has been reported to range between 0.1 and 15% among Caucasians, Hispanics and African Americans [3]. In European Caucasians, the prevalence for AgP has been estimated to be between 0.1 and 1% of the general population [4]. Family studies have indicated an increased prevalence of AgP among siblings with 40-50% being affected. However, the exact mode of inheritance of AgP remains unclear [3].

Coronary heart disease (CHD) is a complex systemic disorder representing the leading cause of death worldwide [5]. As for AgP, it has been known for a long time that CHD is strongly influenced by genetic factors [6], but it was not until recently that genetic risk loci contributing to the development of the disease were identified. An association between CHD and periodontitis has been clearly established [7,8]. Both diseases have several environmental and behavioural risk factors in common such as education, smoking, and diabetes mellitus [8,9]. It is assumed that, in addition to common environmental and behavioural risk factors, this association might also be based on a shared genetic background. Imbalances in immune responses and dysregulation of chronic inflammation are typical attributes of both CHD and periodontitis [10,11]. These similarities point towards the possibility that both diseases have common underlying pathogenic mechanisms, modulated by shared genetic susceptibility loci.

In 2007, four independent GWAS which identified susceptibility loci for CHD were published [12-15]. All studies reported a strong association of a region of elevated linkage disequilibrium (LD) on human chromosome *9p21.3 *with CHD. The identified region is located upstream of the *CDKN2A *and *CDKN2B *genes. A subsequent meta-analysis confirmed this association [16] and the described locus on *9p21.3 *now represents the best replicated genetic CHD risk locus.

Recently, Schaefer *et al*. (2009) [17] investigated the hypothesis that shared susceptibility loci might increase the risk for periodontitis as well as CHD. They analyzed the *9p21.3 *locus for a possible association with AgP in which genetic factors play an important role [18]. Given the postulated higher penetrance of susceptibility genes in AgP compared to chronic periodontitis, this approach was assumed to increase the likelihood to detect statistically significant associations. Schaefer and co-workers [17] genotyped six tagging SNPs located within the *9p21.3 *locus which they subdivided into two defined regions with three tagging SNPs in each. This approach allowed the mapping of the corresponding LD regions (rs2891168, rs1333042, rs1333048 for LD region 1; rs7044859, rs7865618, rs496892 for LD region 2). Genotyping results generated using DNA samples from patients suffering either from localized or generalized AgP were compared to data obtained using DNA samples from control individuals without AgP. Schaefer *et al*. (2009) [17] were able to demonstrate statistically significant associations of all tagging SNPs of LD region 1 and of rs496892 from LD region 2 with localized AgP and even stronger associations with gAgP. The SNPs rs7044859 and rs7865618 did not show any association. The study confirmed the prediction that shared susceptibility genes might be involved in the development of CHD and AgP. However, due to the limited size of the cohorts analyzed (159 generalized AgP patients versus 736 healthy controls and 146 localized AgP patients versus 368 healthy controls) that were small compared to other association studies on complex diseases, the authors emphasized the need for confirmation of the described association in further and/or larger AgP populations.

In the present study, we analyzed a cohort of 130 AgP patients and 339 appropriate periodontally healthy control individuals and genotyped the four tagging SNPs (rs2891168, rs1333042, rs1333048 and rs496892) that had been associated with AgP by Schaefer *et al*. (2009) [17]. Our AgP patient cohort comprised exclusively individuals with gAgP. Patients suffering from this most severe form of AgP showed the strongest association findings in the study of Schaefer and co-workers [17], and therefore, our cohort, exhibiting a similar size as the gAgP cohort in their study, should be adequately suited to confirm their results.

## Methods

### Study Population

The study was limited to Caucasians with a minimum of 20 teeth. In order to ensure a common Caucasian background, only subjects of German or Northern Irish ethnicity were included. The 130 gAgP cases were recruited by outpatient departments throughout Germany (n = 85) and Northern Ireland (n = 45). The gAgP cohort consisted of 53 males (41%) and 77 females (59%). The age at the time of the first diagnosis of gAgP ranged between 15 and 35 years, (inclusion criterion: age ≤ 35 years), the mean age at the time of diagnosis was 30.1 years, the median age 32 years. To be classified with gAgP where full-mouth dental radiographs were available, at least one third of all teeth had to exhibit at least 50% bone loss. In cases where no radiographs were available, a minimum of one third of all teeth had to have clinical attachment loss of ≥ 5 mm.

The 339 control subjects without AgP were also recruited from Germany (n = 225) and Northern Ireland (n = 114). The majority of the German controls (n = 172) were selected from the epidemiological Study of Health in Pomerania (SHIP) [19]. All SHIP participants received an extensive dental examination including assessment of tooth count and location, of probing depth and clinical attachment loss [20]. Information on various risk factors including smoking status, school education and diabetes were obtained through interviews as a part of the medical examination. The remaining German control individuals (n = 53) were recruited by periodontal departments throughout Germany. All Northern Irish controls were participants in the Prospective Epidemiological Study of Myocardial Infarction [21] who had a periodontal examination [22]. The control cohort consisted of 196 males (58%) and 143 females (42%). The age of the control individuals at the time of the dental examination ranged between 40 and 80 years, (exclusion criterion: age < 40 years), the mean age at the time of examination was 55 years, the median age 56.2 years. All controls had to have a minimum of 20 teeth and no clinical attachment loss of ≥ 5 mm. All cases and controls gave their written informed consent for the study. The study was approved by the local institutional review board (Ethics Commission of the Ernst Moritz Arndt University Greifswald, Medical Faculty, registration number BB 05/07). In the current work, the described study population is subsequently referred to as the "Greifswald cohort".

### Genotyping

Genomic DNA was extracted from blood samples using the QIAamp DNA blood mini kit (Qiagen, Hilden, Germany) following the manufacturer's instruction. Purity and concentration of the DNA was determined using a NanoDrop^® ^ND-1000 UV-Vis Spectrophotometer (NanoDrop Technologies, Wilmington, USA), and the integrity of each DNA preparation was verified by gel electrophoresis on 0.8% Agarose-1 × TBE gels stained with ethidium bromide.

Genotyping was performed using an Applied Biosystems 7900HT Fast Real-Time PCR System and the TaqMan Genotyping System (Applied Biosystems, Foster City, CA, USA). All TaqMan assays used in this study were purchased from Applied Biosystems (Darmstadt, Germany) and performed according to the manufacturer's instructions. Genotypes were generated by automatic calling using the SDS 2.3 software (Applied Biosystems). All genotypes were furthermore reviewed manually.

### Statistical Analysis

Unadjusted and adjusted case-control analysis was performed using PLINK v1.06 [23]. We assessed the significance of associations between genotypes and gAgP using logistic regression. The adjusted model was computed by multiple logistic regression analysis adjusting for individual's gender and smoking status. Each individual was classified as never, former or current smoker based on self-reported or interview based information. The smoking status was addressed in the regression model by implementing two dummy variables indicating former and current smoking, with never smokers as the reference. Finally, three covariates were added for adjustment: one for gender and two coding the smoking status.

Meta-analysis was performed in R v2.8.0 http://www.r-project.org using a sample size weighted fixed effect model. To perform the appropriate analyses, we matched the effect alleles of the Greifswald cohort to the effect allele of Schaefer and co-workers (2009) [17]. Additional software and tools used for analyses included Haploview [24], UCSC genome browser http://genome.ucsc.edu, HapMap http://www.hapmap.org and Medline.

## Results

### Minor allele frequencies of the analyzed SNPs rs2891168, rs1333042, rs1333048, and rs496892

Allele frequencies for the minor alleles of all four SNPs genotyped in this study were around 50% (see Table 1). For SNPs rs2891168, rs1333042, and rs1333048 the minor alleles (G, G, and C, respectively) were the same as described by Schaefer and co-workers (2009) [17]. The effect allele of rs496892 was around 50% in our study as well as in the study of Schaefer and co-workers (2009) (A to G) [1]. All four analyzed SNPs were found to be in Hardy-Weinberg-Equilibrium (HWE) in our cohort (p_HWE _> 0.01) (Table 2).

**Table 1:** Allele frequencies in study populations of interest

CHR	SNP ID	Greifswald cohort				Schaefer *et al*. cohort			
		
		A1	A2	FA1	FA2	A1	A2	FA1	FA2
9	rs2891168	G	A	0.47	0.53	G	A	0.46	0.54

9	rs1333042	G	A	0.49	0.51	G	A	0.48	0.52

9	rs1333048	C	A	0.49	0.51	C	A	0.47	0.53

9	rs496892	A	G	0.49	0.51	G	A	0.49	0.51

CHR: Chromosome; A1: Minor allele; A2: Major allele; FA1: Allele frequency of the minor allele in the respective cohort; FA2: Allele frequency of the major allele in the respective cohort.

**Table 2:** Hardy-Weinberg-Equilibrium of all SNPs analyzed in the Greifswald cohort

CHR	SNP ID	HWE TEST	A1	A2	GENO	O(HET)	E(HET)	HW p-value
9	rs2891168	**ALL**	G	A	108/229/132	0.49	0.50	0.64

9	rs2891168	**AFF**	G	A	34/69/27	0.53	0.50	0.60

9	rs2891168	**UNAFF**	G	A	74/160/105	0.47	0.50	0.38

9	rs1333042	**ALL**	G	A	111/231/125	0.49	0.50	0.85

9	rs1333042	**AFF**	G	A	35/68/27	0.52	0.50	0.72

9	rs1333042	**UNAFF**	G	A	76/163/98	0.48	0.50	0.59

9	rs1333048	**ALL**	C	A	114/229/126	0.49	0.50	0.64

9	rs1333048	**AFF**	C	A	38/65/27	0.50	0.50	1.00

9	rs1333048	**UNAFF**	C	A	76/164/99	0.48	0.50	0.66

9	rs496892	**ALL**	T	C	99/257/113	0.55	0.50	0.04

9	rs496892	**AFF**	T	C	21/68/41	0.52	0.49	0.48

9	rs496892	**UNAFF**	T	C	78/189/72	0.56	0.50	0.04

CHR: Chromosome; HWE Test: Test of the respective SNP for Hardy-Weinberg Equilibrium for all analyzed individuals (ALL), cases with gAgP (AFF), and control subjects unaffected by gAgP (UNAFF); A1: Minor allele; A2: Major allele; GENO: Genotype counts: Number of individuals homozygous for the minor allele/Number of heterozygous individuals/Number of individuals homozygous for the major allele; O(HET): Observed heterozygosity; E(HET): Expected heterozygosity; HW p-value: Hardy-Weinberg p-value.

### Analysis for Association with gAgP using unadjusted SNP data

Dominant, recessive, and multiplicative models were used to test for associations with gAgP in both unadjusted and adjusted SNP data. The results for the unadjusted analyses are summarized in Table 3. Without adjustment, rs2891168 was significantly associated with gAgP in a dominant and a multiplicative model, respectively, [p = 2.89 × 10^-02^, OR = 1.71 (1.06-2.77) and p = 4.92 × 10^-02^, OR = 1.33 (1.00-1.77)], demonstrating stronger association in the dominant model. In contrast, in the case of rs1333042, none of the three genetic models produced values below the significance threshold. The two SNPs rs1333048 and rs496892 were both significantly associated with gAgP in a multiplicative model [p = 3.95 × 10^-02^, OR = 1.35 (1.02-1.80) and p = 1.38 × 10^-02^, OR = 1.47 (1.09-2.00), respectively], but only rs496892 did also show significant association in the dominant model [p = 2.03 × 10^-02^, OR = 1.71 (1.09-2.70)]. Using a recessive model, none of the four SNPs showed a significant association with gAgP.

**Table 3:** Associations of the four candidate SNPs with generalized Aggressive Periodontitis (gAgP) in the Greifswald cohort

		Recessive model			Multiplicative model			Dominant model		
	
	SNP ID	P	OR	95% CI	P	OR	95% CI	P	OR	95% CI
**Unadjusted analysis**	**rs2891168**	3.20 × 10^-01^	1.27	0.79-2.03	4.92 × 10^-02^	1.33	1.00-1.77	**2.89 × 10^-02^**	**1.71**	**1.06-2.77**
	
	**rs1333042**	3.21 × 10^-01^	1.27	0.80-2.01	8.46 × 10^-02^	1.29	0.97-1.71	7.04 × 10^-02^	1.56	0.96-2.54
	
	**rs1333048**	1.25 × 10^-01^	1.43	0.91-2.26	**3.95 × 10^-02^**	**1.35**	**1.02-1.80**	6.64 × 10^-02^	1.57	0.97-2.55
	
	**rs496892**	1.05 × 10^-01^	1.55	0.90-2.63	**1.38 × 10^-02^**	**1.47**	**1.09-2.00**	2.03 × 10^-02^	1.71	1.09-2.70

**Adjusted analysis**	**rs2891168**	3.18 × 10^-01^	1.303	0.77-2.19	6.44 × 10^-02^	1.35	0.98-1.85	**4.73 × 10^-02^**	**1.70**	**1.01-2.88**
	
	**rs1333042**	2.88 × 10^-01^	1.321	0.79-2.21	9.51 × 10^-02^	1.31	0.95-1.80	9.99 × 10^-02^	1.56	0.92-2.64
	**rs1333048**	1.18 × 10^-01^	1.498	0.90-2.49	**4.22 × 10^-02^**	**1.39**	**1.01-1.91**	7.95 × 10^-02^	1.60	0.95-2.72
	**rs496892**	2.20 × 10^-01^	1.43	0.81-2.55	**4.51 × 10^-02^**	**1.41**	**1.01-1.98**	4.94 × 10^-02^	1.65	1.00-2.72

Association statistics are shown for cases consisting of individuals affected with gAgP and controls consisting of individuals unaffected by gAgP. OR: Odds Ratio; 95% CI: 95% Confidence Intervals; P: p-values obtained by logistic regression using PLINK v1.06 [23]. Values are given before and after adjustment for the covariates smoking and gender. The parameters for the genetic models producing the strongest significant associations are given in boldface for each SNP for adjusted as well as unadjusted analyses.

### Analysis for Association with gAgP using adjusted SNP data

In the next step, analyses were adjusted for the covariates gender and smoking. Using the multiplicative model, the SNPs rs1333048 and rs496892 exhibited a statistically significant association with gAgP, whereas in the dominant model, only rs2891168 and rs496892 demonstrated significant associations. Again, as was the case with the unadjusted data, rs1333042 did not show significant association with gAgP in any of the three models. Furthermore, in the recessive model, none of the four SNPs was significantly associated with gAgP (Table 3).

Taken together, two of the SNPs of the LD region 1, (rs2891168 and rs1333048) demonstrated significant association with gAgP in the multiplicative model, whereas only rs2891168 was also significantly associated in the dominant model. The association of rs2891168 with gAgP was stronger in the dominant than the multiplicative model, using both unadjusted data [p = 2.89 × 10^-02^, OR = 1.71 (1.06-2.77) versus p = 4.92 × 10^-02^, OR = 1.33 (1.00-1.77)] and adjusted data [p = 4.73 × 10^-02^, OR = 1.70 (1.01-2.88) versus p = 6.44 × 10^-02^, OR = 1.35 (0.98-1.85)]. In the case of rs1333048, using unadjusted as well as adjusted data, the multiplicative model produced similar significant associations [p = 1.38 × 10^-02^, OR = 1.47 (1.09-2.00) and p = 4.22 × 10^-02^, OR = 1.39 (1.01-1.91)]. The SNP rs496892 that represented LD region 2 was significantly associated with gAgP in the multiplicative and in the dominant model using unadjusted as well as adjusted data, with the association stronger in both cases when the multiplicative model was used (Table 3).

### Meta-Analysis for Association with gAgP using all available data for the SNPs rs2891168, rs1333042, rs1333048, and rs496892

To maximize the statistical power of the association analyses, we finally performed a meta-analysis for the SNPs rs2891168, rs1333042, rs1333048, and rs496892 combining our adjusted data with those from Schaefer and co-workers (2009) [17] for multiplicative, recessive, and dominant models. In the multiplicative model, all four tested SNPs were significantly associated with gAgP, even after Bonferroni correction (see Table 4). In this model, rs1333048 exhibited the strongest association with p = 1.09 × 10^-03^. For all four SNPs, the meta-analysis produced lower p-values in the multiplicative model compared to the results obtained using the genotyping data from the two single cohorts. Using a recessive model in the meta-analysis, only the SNPs representing LD region 1 (rs2891168, rs1333042, rs1333048) were significantly associated with gAgP, whereas rs496892 representing LD region 2 did not fall below the threshold of significance. As in the case of the multiplicative model, rs1333048 showed the strongest association with gAgP with p = 9.97 × 10^-04^. However, whereas in the multiplicative model the p-values produced in the meta-analysis were lower than those obtained with the two single cohorts, this was not the case in the recessive one: here, the p-values from the meta-analysis, while being predominantly lower compared to those obtained solely with the Greifswald cohort, were higher than the values obtained when only the Schaefer *et al*. cohort was used. Using a dominant model in the meta-analysis, only rs496892 representing LD region 2 exhibited significant association after Bonferroni correction with p = 1.24 × 10^-02^, with this value being lower than those obtained using the two single cohorts (p = 4.94 × 10^-02 ^and p = 2.60 × 10^-02 ^for the Greifswald and the Schaefer *et al*. cohort [17], respectively).

**Table 4:** Meta-Analysis for Association of the four candidate SNPs with generalized Aggressive Periodontitis (gAgP) using the adjusted data from the Greifswald cohort and those published by Schaefer and co-workers [17]

	SNP ID	P Greifswald cohort	n Greifswald cohort	P Schaefer *et al*. cohort	n Schaefer *et al*. cohort	P Meta-Analysis	P (BON) Meta-Analysis
**Recessive model**	rs2891168	3.18 × 10^-01^	468	**8.80 × 10^-04^**	879	1.06 × 10^-03^	4.22 × 10^-03^
	
	rs1333042	2.88 × 10^-01^	466	**1.60 × 10^-03^**	879	1.49 × 10^-03^	5.95 × 10^-03^
	
	rs1333048	1.18 × 10^-01^	468	**6.90 × 10^-04^**	876	**2.49 × 10^-04^**	**9.97 × 10^-04^**
	
	rs496892	2.20 × 10^-01^	468	1.50 × 10^-01^	862	5.92 × 10^-02^	2.37 × 10^-01^

**Multiplicative** **model**	rs2891168	6.44 × 10^-02^	468	4.40 × 10^-03^	879	**6.97 × 10^-04^**	**2.79 × 10^-03^**
	
	rs1333042	9.51 × 10^-02^	466	4.80 × 10^-03^	879	**1.10 × 10^-03^**	**4.42 × 10^-03^**
	
	rs1333048	**4.22 × 10^-02^**	468	2.50 × 10^-03^	876	2.73 × 10^-04^	1.09 × 10^-03^
	
	rs496892	**4.51 × 10^-02^**	468	**2.40 × 10^-02^**	862	**2.65 × 10^-03^**	**1.06 × 10^-02^**

**Dominant model**	rs2891168	**4.73 × 10^-02^**	468	1.40 × 10^-01^	879	1.82 × 10^-02^	7.28 × 10^-02^
	
	rs1333042	9.99 × 10^-02^	466	1.20 × 10^-01^	879	2.60 × 10^-02^	1.04 × 10^-01^
	
	rs1333048	7.95 × 10^-02^	468	9.60 × 10^-02^	876	1.74 × 10^-02^	6.95 × 10^-02^
	
	rs496892	4.94 × 10^-02^	468	2.60 × 10^-02^	862	3.10 × 10^-03^	1.24 × 10^-02^

Association statistics are shown for the Greifswald total cohort consisting of cases affected with gAgP and control subjects unaffected by gAgP and for the gAgP cases and the corresponding controls described in Schaefer *et al*. (2009) [17]. P: p-values obtained by logistic regression using PLINK; n: Number of individuals in the corresponding cohorts; P Meta-Analysis and P (BON) Meta-Analysis: p-values of the Meta-Analyses using z statistics before and after Bonferroni correction. Values are given after adjustment for the covariates smoking and gender. The parameters for the genetic models producing the strongest significant associations are given in boldface for each SNP before and after Bonferroni correction.

In summary, the lowest p-values in total were obtained for rs2891168, rs1333042, and rs496892 when the multiplicative model was used, and in the case of rs1333048, the value was only marginally lower in the recessive (p = 9.97 × 10^-04^) as compared to the multiplicative model (p = 1.09 × 10^-03^). Therefore, the multiplicative model generated the most consistent results and appeared to be the most plausible model for the association of all four tested SNPs with gAgP.

## Discussion

**Figure 1:**
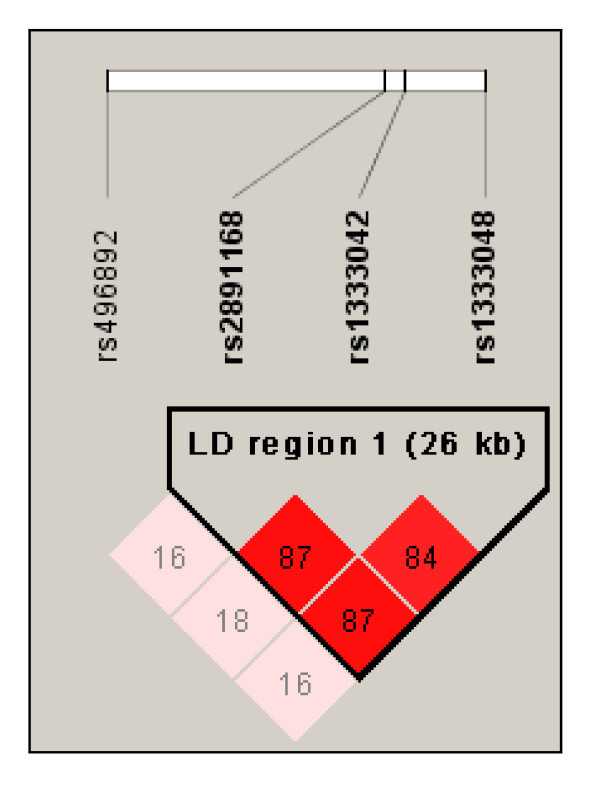
**Linkage disequilibrium (LD) map for the SNPs genotyped in this study**. The LD region 1 as described by Schaefer *et al*. (2009) [17] is represented by the three SNPs rs2891168, rs1333042, and rs1333048. The LD region 2 is represented by SNP rs496892. The derived LD block structure is shown for the Greifswald case-control cohort (n = 469) described in this study. Numbers represent pair wise percent R^2^-values.

The main finding of the current study was the replication of statistically significant associations between three defined SNPs located on human chromosome *9p21.3 *and gAgP. The locus on chromosome *9p21.3*, which had previously been shown to be associated with CHD, was recently associated with gAgP (Figure 1) [17]. The replication analysis in our independent cohort, consisting of 130 gAgP patients and 339 periodontally healthy control subjects without gAgP, strengthened the findings of Schaefer *et al*. (2009) [17]. This result corroborates the idea that the chromosomal locus on *9p21.3 *may represent one of the postulated genetic links between CHD and periodontitis. Furthermore, combining the results of Schaefer *et al*. and our own results in a meta-analysis resulted in an improved statistical power due to the increase in the total number of analyzed individuals. The results of this meta-analysis favour a multiplicative model of inheritance for the genetic variant that is associated with gAgP.

**Figure 2:**
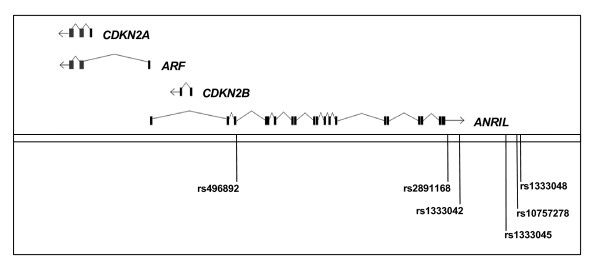
**Transcriptional map of the human *9p21.3 *locus associated with CHD and gAgP**. The transcripts specifying *CDKN2A*, *CDKN2B*, *ARF*, and *ANRIL *are shown as well as the four SNPs tested in this study (rs496892, rs2891168, rs1333042, rs1333048) and the two SNPs rs10757278 and 1333045 analyzed extensively in the studies of [26] and [25], respectively (illustration adopted from [26] and modified, by courtesy of Norman E.  Sharpless).

Detailed functional analyses of the *9p21.3 *region have been completed (Figure 2) to determine the molecular mechanisms underlying the increased risk for CHD [25,26]. Within this locus, the non-coding *ANRIL *RNA overlaps with the upstream promoter region of the genes *CDKN2A *and *ARF*, whereby *ARF *shares two exons with *CDKN2A*, and the complete sequence of the *CDKN2B *gene. While *CDKN2A*, *ARF*, and *CDKN2B *are transcribed in the same direction, *ANRIL *is transcribed in the opposite direction (Figure 2). Both *CDKN2A *and *CDKN2B *encode cyclin-dependent kinase inhibitors, namely *p16*^INK4a ^and *p15*^INK4b^. The gene product of *ARF *is able to bind and sequester MDM1, a protein responsible for the degradation of p53, and so acts as a stabilizer of the tumor suppressor protein p53. Therefore, these three genes specify proteins involved in cell cycle inhibition.

Liu *et al*. (2009) [26] analyzed the expression of several transcripts specified by the *9p21.3 *region in purified peripheral blood T-cells (PBTL) from 170 healthy donors. These samples were genotyped for six selected disease-related SNPs spanning the locus. The PBTL of individuals that harbored a common SNP (rs10757278) had significantly reduced expression of all transcripts encoded by the *9p21.3 *region, namely *CDKN2A*, *CDKN2B*, *ARF *and *ANRIL*. The SNP (rs10757278) has been associated with an increased risk of CHD, stroke and aortic aneurysm and is located downstream of the 3'-end of *ANRIL *(Figure 2). Therefore, genotypes of rs10757278 which are associated with an increased risk of atherosclerotic diseases are also associated with a decreased expression of genes *CDKN2A*, *CDKN2B *and *ARF*, which encode products with known anti-proliferative functions, and, in addition, with a reduced expression of *ANRIL *itself [26]. Based on their findings, Liu and co-workers suggested a potentially important role for cell cycle inhibitors in atherosclerotic disease. According to a regulation model derived from these results, a polymorphic *cis*-regulatory element in close linkage disequilibrium with rs10757278 influences the expression of *CDKN2A*, *CDKN2B *and *ARF *which are located approximately 120 kb from this SNP as well as of *ANRIL *whose 3'-end is located in the direct neighborhood of rs10757278 and which spans several other risk-associated SNPs (Figure 2). Regulation of *CDKN2A*, *CDKN2B *and *ARF *could be directly mediated by the postulated polymorphic *cis*-regulatory element or, as *ANRIL *overlaps with the upstream region of the *CDKN2A *and *ARF *genes and the complete sequence of *CDKN2B*, indirectly *via ANRIL *[26].

Jarinova and co-workers (2009) [25] performed an even more detailed functional analysis of the *9p21.3 *region in an attempt to identify the molecular mechanisms responsible for the increased risk for CHD. It turned out that the risk haplotype can finally be reduced to one single nucleotide variation, namely rs1333045, which seems to be responsible for an increased expression of the short splicing variants of the *ANRIL *regulatory RNA in homozygous carriers of the risk allele as compared to carriers of the non-risk allele, whereas the long *ANRIL *variant is present in decreased amounts [25]. Jarinova and co-workers were able to demonstrate a positive correlation between the long splicing variant of *ANRIL *and the amount of *CDKN2A *and *CDKN2B *specific mRNA. On the other hand, the amount of *CDKN2A *and *CDKN2B *specific mRNA is reduced in the homozygous carriers of the risk allele who exhibited decreased amounts of the long *ANRIL *variant and increased amounts of the short splicing variants. The authors hypothesized that the SNP(s), such as rs1333045, within the *9p21.3 *locus may function as a molecular switch resulting in reciprocal changes in expression levels of the short and long *ANRIL *transcripts. According to this model, the risk allele causes increased amounts of short *ANRIL *variants, which in turn results in reduced expression of *CDKN2A *and *CDKN2B *(and *ARF*). As *CDKN2A*, *CDKN2B *and *ARF *encode proteins involved in negative cell cycle regulation their down-regulation could be predicted to promote a pro-proliferative phenotype. Indeed, genome-wide expression profiling performed by Jarinova and co-workers (2009) [25] using whole-blood RNA demonstrated up-regulation of gene sets modulating cellular proliferation in homozygous carriers of the risk allele. Of particular importance in the context of gAgP, gene sets relevant to inflammation, including TNF/MAP kinase pathway signaling in activated T-cells and interferon response genes turned out to be differentially expressed. It is therefore reasonable to suggest that the same molecular mechanisms involving the SNP rs1333045 that result in an increased risk for CHD could also mediate the increased risk for gAgP.

**Figure 3:**
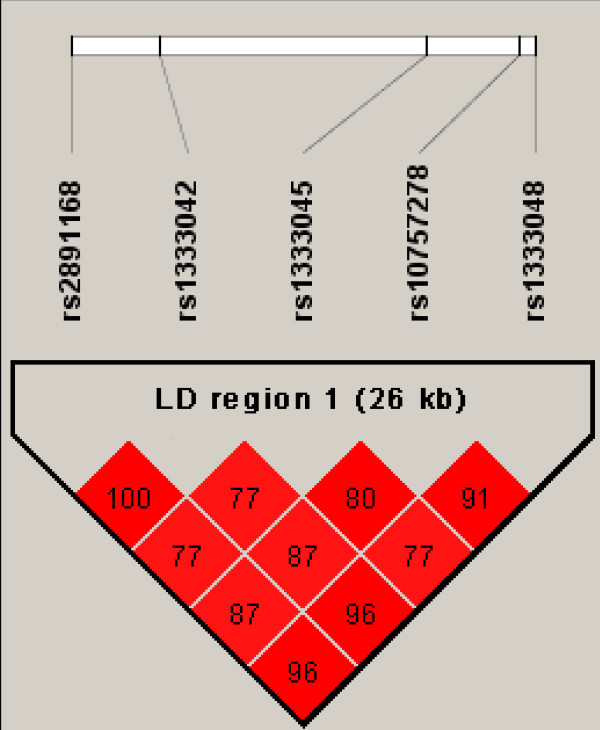
**Linkage disequilibrium (LD) map for the three SNPs within LD region 1 genotyped in this study (rs2891168, rs1333042, and rs1333048) as well as for rs10757278 and the putative causative SNP rs1333045**. The derived LD block structure is derived from the HAPMAP data. Numbers represent pair wise percent R^2^-values.

The SNPs rs1333045 and rs10757278, which were extensively investigated [25,26], are located within the LD region 1 between rs2891168 and rs1333048 that have been genotyped in the current study (Figure 2). The chromosomal order of the three SNPs in the LD region 1 which were genotyped in our study (rs2891168, rs1333042, and rs1333048) as well as rs1333045 and rs10757278 and the corresponding R^2 ^values as calculated using the HAPMAP data are shown in Figure 3. The high values clearly confirm the LD structure of the region. Using the available HAPMAP data for these five SNPs, a haplotype structure which also considers the newly identified causative SNP rs1333045 and rs10757278 was calculated (Table 5). Altogether six haplotypes were obtained, and two (AATAA and GGCGC) accounted for more than 92% of all haplotypes detected at this locus according to the HAPMAP data. Three of these (GGCGC, AACAA, and AACGC), which together amounted to 53.5% of all haplotypes identified, exhibit the risk allele C of the putative causative SNP rs1333045. Obviously, the risk allele is quite frequent in the Caucasian population.

**Table 5:** Identified Haplotypes for LD region 1 based on the SNPs rs2891168, rs1333042, rs1333045, rs10757278, and rs1333048 as derived from the HAPMAP data

Derived Haplotype	Total Haplotype Frequency
GG**C**GC	0.480

AATAA	0.441

AA**C**AA	0.050

GGTAC	0.020

AA**C**GC	0.005

AATGC	0.005

The five SNPs rs2891168, rs1333042, rs1333045, rs10757278, and rs1333048 were used to determine the minimal number of haplotypes and the corresponding frequencies present in LD region 1 as derived from the HAPMAP data set. The postulated causative C residue within the three putative risk haplotypes is shown in boldface.

The number of gAgP cases in our case-control cohort was similar to that analyzed by Schaefer and co-workers (n = 130 versus n = 159), but the number of control individuals was clearly smaller in our cohort (n = 339 versus n = 736). However, the information about the periodontal condition of the control subjects described as "free of periodontitis" in the cohort of Schaefer and co-workers was based solely on self-report of their oral health status. In contrast, our control subjects were classified to be free of even low levels of periodontitis based on an extensive dental examination. Therefore, the more stringent ascertainment criteria for inclusion in our control cohort should compensate for the smaller number of individuals.

Most importantly, the combination of our genotyping results with those from Schaefer and co-workers in a meta-analysis for the SNPs rs2891168, rs1333042, rs1333048, and rs496892 produced lower p-values as compared to the results of both the individual studies in the multiplicative model. It seems reasonable to propose a multiplicative model for the whole locus covering both LD regions, substantiating the earlier assumption of Schaefer and co-workers that the underlying genetic model might be "somewhere between the recessive and the multiplicative one" [17].

The association data support the hypothesis which predicts increased susceptibility for the development of gAgP determined by the risk haplotype associated with a pronounced general pro-proliferative and pro-inflammatory phenotype resulting from decreased expression of the three cell-cycle inhibitor encoding genes *CDKN2A*, *ARF *and *CDKN2B*.

The chromosomal region *9p21.3 *probably does not represent the sole susceptibility locus for the development of gAgP. The predicted risk haplotypes, as calculated from the CEU HAPMAP data, amount to a total frequency of 53.5%, which is surprisingly high in view of the known low prevalence rate of gAgP. Therefore, in addition to the influence of lifestyle factors, it can be speculated that further genetic loci for gAgP exist. These loci might exhibit stronger gene related effects in their contribution to the increased risk for the development of gAgP. A hypothesis-free Genome-wide Association Study (GWAS) could represent the method of choice to identify such predicted additional gAgP susceptibility genes. As a clinical implication of the verified association between gAgP and the chromosomal region *9p21.3 *that has been clearly associated with CHD in earlier studies, it may be worth informing AgP patients routinely of a possible increased risk of CHD.

## Conclusions

In conclusion, using an independent case-control cohort, we positively replicated the finding of an association between the chromosomal region *9p21.3 *and gAgP which was first described by Schaefer and co-workers [17]. This result strengthens support for the hypothesis that shared susceptibility genes within this chromosomal locus might be involved in the pathogenesis of both CHD and gAgP.

## Competing interests

The authors declare that they have no competing interests.

## Authors' contributions

FDE participated in the molecular genetic analyses and in drafting the manuscript and confirmed the quality of the genetic data. KU participated in the molecular genetic analyses. AT carried out all statistical analyses. JF, SS, BN, JG, SR, PE, and GJL were involved in sample and data collection as well as in phenotype data quality control and multi-centric study coordination. BH and PM participated in the design and coordination of the study. GH and TK conceived and designed the study and revised the manuscript. All authors read and approved the final manuscript.

## Pre-publication history

The pre-publication history for this paper can be accessed here:

http://www.biomedcentral.com/1471-2350/11/119/prepub
